# Use of pre-industrial baselines to monitor anthropogenic enrichment of metals concentrations in recently deposited sediment of floodplain lakes in the Peace-Athabasca Delta (Alberta, Canada)

**DOI:** 10.1007/s10661-020-8067-y

**Published:** 2020-01-10

**Authors:** Tanner J. Owca, Mitchell L. Kay, Jelle Faber, Casey R. Remmer, Nelson Zabel, Johan A. Wiklund, Brent B. Wolfe, Roland I. Hall

**Affiliations:** 1 Department of Geography and Environmental Studies, Wilfrid Laurier University, 75 University Avenue West, Waterloo, Ontario N2L 3C5 Canada; 2 Department of Biology, University of Waterloo, Waterloo, Ontario N2L 3G1 Canada

**Keywords:** Wood Buffalo National Park, River sediment quality, Alberta oil sands, Aquatic ecosystem monitoring, Environmental impact assessment, Paleolimnology

## Abstract

Well-designed monitoring approaches are needed to assess effects of industrial development on downstream aquatic environments and guide environmental stewardship. Here, we develop and apply a monitoring approach to detect potential enrichment of metals concentrations in surficial lake sediments of the Peace-Athabasca Delta (PAD), northern Alberta, Canada. Since the ecological integrity of the PAD is strongly tied to river floodwaters that replenish lakes in the delta, and the PAD is located downstream of the Alberta oil sands, concerns have been raised over the potential transport of industry-supplied metals to the PAD via the Athabasca River. Surface sediment samples were collected in September 2017 from 61 lakes across the delta, and again in July 2018 from 20 of the same lakes that had received river floodwaters 2 months earlier, to provide snapshots of metals concentrations (Be, Cd, Cr, Cu, Ni, Pb, V, and Zn) that have recently accumulated in these lakes. To assess for anthropogenic enrichment, surficial sediment metals concentrations were normalized to aluminum and compared to pre-industrial baseline (i.e., reference) metal-aluminum linear relations for the Athabasca and Peace sectors of the PAD developed from pre-1920 measurements in lake sediment cores. Numerical analysis demonstrates no marked enrichment of these metals concentrations above pre-1920 baselines despite strong ability (> 99% power) to detect enrichment of 10%. Measurements of river sediment collected by the Regional Aquatics- and Oil Sands-Monitoring Programs (RAMP/OSM) also did not exceed pre-1920 concentrations. Thus, results presented here show no evidence of substantial oil sands-derived metals enrichment of sediment supplied by the Athabasca River to lakes in the PAD and demonstrate the usefulness of these methods as a monitoring framework.

**Electronic supplementary material:**

The online version of this article (10.1007/s10661-020-8067-y) contains supplementary material, which is available to authorized users.

## Introduction

As large-scale mining operations continue to expand across northern Canada, so do concerns about releases of contaminants and their effects on downstream aquatic ecosystems (Schindler and Smol 2006; Smol 2008; Schindler 2010). Comprehensive monitoring programs are needed to inform stakeholders, ensure industrial compliance, and guide environmental stewardship decisions that protect these ecosystems from harmful effects of industrial pollution. Monitoring programs may vary depending on the industry or project type, but they all share a common objective to identify anthropogenic effects on the environment (Roach and Walker 2017). However, monitoring efforts are often initiated only after concerns have been raised (Blais et al. 2015). This presents challenges for formulation of evidence-based recommendations by policy-makers, because an absence of sufficient long-term, pre-development data impairs the ability to discern the role of anthropogenic activities from natural processes occurring in the landscape (Smol 2008; Blais et al. 2015). Thus, effectiveness of monitoring programs is greatly improved when they include long-term pre-development data to define baseline (or reference) conditions and the range of natural variation (Smol 1992; Lindenmayer and Likens 2009; Dowdeswell et al. 2010).

In northern Alberta (Canada), local, national, and international concerns have been raised about environmental consequences of contaminant releases from oil sands mining and processing activities to the Athabasca River and, at its terminus, the Peace-Athabasca Delta (PAD). The PAD is one of the world’s largest inland freshwater deltas, and its abundant shallow lakes provide habitat for a variety of biota and resources that support indigenous communities based in Fort Chipewyan. Mostly protected within the boundaries of Wood Buffalo National Park (WBNP), the PAD has gained recognition as a Ramsar Wetland of International Importance and contributed to the listing of WBNP as a UNESCO World Heritage Site for its historical, ecological, and cultural significance. Shallow lakes dominate this landscape and are largely dependent on periodic ice-jam flood events that occur along the Peace and Athabasca rivers to maintain their water balances (PADPG 1972; Prowse and Conly 1998; Wolfe et al. 2007; Timoney 2013; Remmer et al. 2018). Although river floodwaters play an integral part in lake hydrological and ecological conditions, they also convey sediment and associated substances of concern from upstream sources, including metals from oil sands operations. Bitumen deposits in the Alberta oil sands region, ~ 200 river-km upstream of the PAD, are the largest and shallowest among Canada’s reserves (Dowdeswell et al. 2010). Here, the Athabasca River and some of its major tributaries flow through the deposits situated along the river banks and naturally erode bitumen exposures and release associated metals. It is therefore essential to identify the natural range of metals concentrations in the Athabasca River to accurately evaluate the extent of river pollution contributed by oil sands operations to the PAD.

It has remained challenging to assess the extent of metals enrichment at the PAD because systematic monitoring in the Lower Athabasca Oil Sands Region by the industry-funded Regional Aquatics Monitoring Program (RAMP) was not initiated until 1997—three decades after the start of oil sands production (Cronmiller and Noble 2018). The lack of pre-industrial baseline data has impeded ability of RAMP and other subsequent monitoring programs to detect and quantify the extent to which the industry has increased supply of substances of concern to the Athabasca River and downstream delta. Indeed, these programs have been criticized for their study design and inability to detect trends of contaminant concentration since onset of industrial development (e.g., Dowdeswell et al. 2010; Gosselin et al. 2010; Schindler 2010; Dillon et al. 2011). This has resulted in calls for better monitoring practices for nearly a decade and the emergence of a petition in 2014, led by the Mikisew Cree First Nation (MCFN) to the World Heritage Committee (WHC) and International Union for the Conservation of Nature (IUCN) to add WBNP to the List of World Heritage in Danger. Among several concerns, this petition cited oil sands development as an immediate threat to the integrity of WBNP (MCFN 2014). In response, WHC/IUCN (2017) outlined 17 recommendations for WBNP to address in their Reactive Monitoring Mission Report. Recommendation #9 states the need to “expand the scope of monitoring and project assessments to encompass possible individual and cumulative impacts on the Outstanding Universal Value of the property and in particular the PAD” (WHC/IUCN 2017, p. 4). In reply, WBNP (2019) stated in the Action Plan that the Oil Sands Monitoring (OSM) program, led by the federal and provincial governments, is currently assessing the cumulative impacts of oil sands operations on the PAD. In the OSM’s 2017–2018 annual report, their first listed objective was to obtain data on baseline conditions (OSM 2018). Knowledge of pre-industrial baseline conditions has remained a key and fundamental knowledge gap for monitoring programs, lingering since the 2011 Integrated Oil Sands Environment Monitoring Plan, which was intended to provide guidance to the OSM program (Wrona et al. 2011).

For nearly a decade, paleolimnological analysis of sediment cores from floodplain lakes has been proposed as a promising method to establish pre-industrial baseline concentrations of substances of concern conveyed by the Athabasca River (Dowdeswell et al. 2010; Wrona and di Cenzo 2011). Sediment accumulated by floodplain lakes provides an archive of materials supplied via multiple pathways, including from river floodwaters and the atmosphere, and stratigraphic analyses enable detection of changes in concentrations of substances of concern from natural and anthropogenic sources (Smol 1992; Wiklund et al. 2012, 2014; MacDonald et al. 2016). This approach was applied by Wiklund et al. (2012, 2014) in the PAD to quantify sediment metals concentrations before onset of oil sands industrial development, which were otherwise unattainable. They represent the first assessments of temporal changes in metals concentrations, via atmospheric and river pathways, at the PAD using pre-industrial baseline conditions established from analyses of radiometrically dated sediment cores. Analysis of a sediment core from a lake elevated above the PAD floodplain reveals that oil sands operations have not yet elevated atmospheric supply of metals to this region, located some 200 km to the north (Wiklund et al. 2012).

Mining-related substances of concern may also enter lakes of the PAD via river floodwaters. Using pre-industrial (defined as pre-1920) baselines derived from analyses of sediment cores from two floodplain lakes, Wiklund et al. (2014) assessed metals concentrations in samples of surficial river-bottom sediment collected by RAMP in the Athabasca Delta. Their study demonstrated that the metals concentrations remain within the range of natural variability. The knowledge of pre-industrial baseline metals concentrations provided useful insight and enabled evaluation of RAMP-collected river sediment for the first time for evidence of industry-caused enrichment. While Wiklund et al. (2014) demonstrated that such approaches could be utilized to interpret metal concentrations in surficial sediment in the Athabasca River and its distributaries, the study was localized to the Athabasca Delta and due to the dynamic nature of fluvial environments, it remains uncertain if the *recently collected* surface sediment that were sampled from the Athabasca River and its distributaries represents *recently deposited* sediment. Flood-prone lakes, on the other hand, accrete sediment vertically and likely serve as a more informative monitor of recently deposited river-derived sediment metals concentrations than river-bottom sediment.

The objective of this study is to assess metals concentrations in recently deposited sediment in a large suite of flood-prone lakes from the PAD for evidence of enrichment using baseline metals concentrations established from the analysis of pre-1920 sediments in cores from six lakes. To accomplish this, surface sediment was sampled in 2017 from 61 lakes, which span the hydrological gradient of lake water balance conditions across the Peace and Athabasca sectors of the PAD (Wolfe et al. 2007). In the following year, timely occurrence of a spring ice-jam flood prompted re-sampling of a subset of these lakes that had flooded to assess metals concentrations in newly deposited sediment supplied from the Peace and Athabasca rivers. RAMP and OSM river-bottom sediment metals concentrations were also assessed using the pre-1920 baseline information derived from lake sediment cores. Metals analyzed in this study include seven priority pollutants listed under the US Environmental Protection Agency’s Clean Water Act (beryllium (Be), cadmium (Cd), chromium (Cr), copper (Cu), lead (Pb), nickel (Ni), and zinc (Zn)), as well as vanadium (V). These eight metals were chosen since a study by Kelly et al. (2010) showed they occur at higher concentrations within the winter snowpack near the oil sands development and in downstream river waters, and were also the suite of metals reported in Wiklund et al. (2014). It is envisioned that the methods used and the framework developed in this study can be adopted by agencies and stakeholders implementing Wood Buffalo National Park’s Action Plan (WBNP 2019) for ongoing monitoring of metals deposition in lakes of the PAD.

## Methods

### Study area

**Fig. 1:**
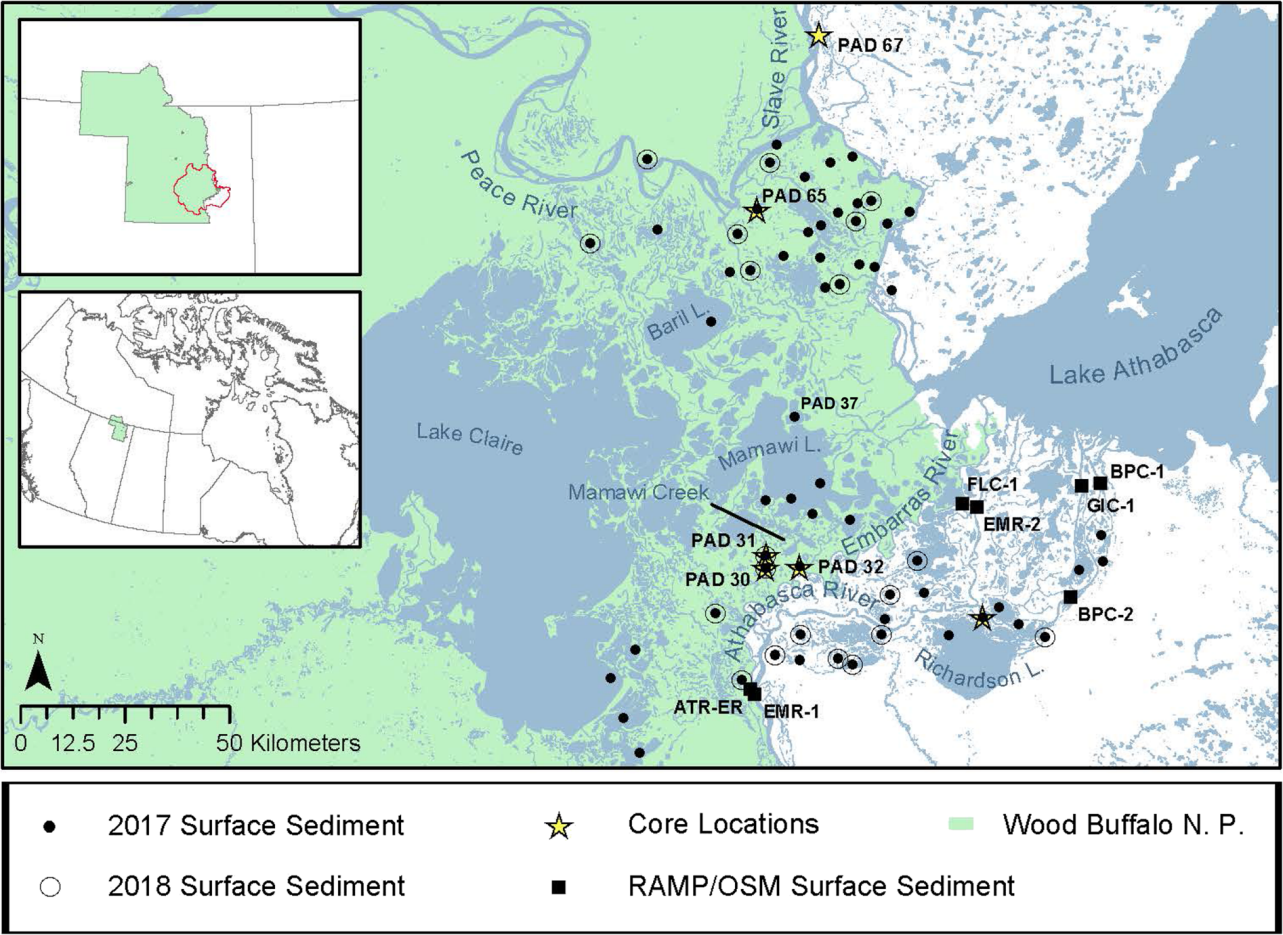
Map of the Peace-Athabasca Delta (PAD) and sampling locations. Lake labels are referred to in the text

Water balance of the abundant, shallow lakes in the PAD is influenced by precipitation and runoff, evaporation, and inflow from rivers, which ranges from continuous to episodic (Wolfe et al. 2007; Fig. 1). Magnitude and frequency of river flooding to lakes varies with elevation and proximity to the river channel network, which cause lakes in the PAD to span a broad hydrological gradient. Based on these varying factors, lakes have been previously designated into three main hydrological categories (PADPG 1972; Pietroniro et al. 1999; Wolfe et al. 2007). Lakes receiving (near-) continuous river through-flow are categorized as open drainage, those periodically receiving river floodwaters during open-water and ice-jam flooding are distinguished as restricted drainage, and lakes that episodically receive river inputs during ice-jam flooding are deemed closed drainage. Lakes located in the northern Peace sector, which are mostly closed drainage, occasionally receive river inputs from the Peace River during ice-jam events, while lakes in the southern Athabasca sector, which are predominantly restricted drainage with few open- and closed- drainage lakes, receive more frequent floodwaters from the Athabasca River during both the spring melt and open-water seasons.

River floodwaters convey suspended sediments that are mineral-rich, but which vary spatially and temporally in grain size. River sediment supplied to floodplain lakes in the Peace and Athabasca sectors of the PAD likely differ in metals signatures, because the Peace and Athabasca rivers flow through different geology. The Peace River flows through sedimentary exhalative deposits (rich in Ag, Cu, Pb, and Zn) and black shale polymetallic deposits (rich in Mo, Ni, and Zn) near the town of Peace River, Alberta (Rukhlov 2011). The Peace River also flows through Devonian carbonate outcrops rich in Zn near Vermilion Chutes, Alberta (Rice 2003; Pana 2003; Rukhlov 2011). North of Fort McMurray, the Athabasca River flows through bitumen deposits containing relatively high concentrations of Ni and V (Speight 2005). The Athabasca River also flows through several Mississippi Valley-Type and Prairie-Type deposits (rich in Au, Ag, platinum group elements, and Cu) between Fort McMurray and the PAD (Rukhlov 2011). The study design outlined in this paper encompasses the spatial extent required to include lakes in the PAD that span the entirety of the hydrological gradient and their potential metal sources.

### Lake sediment core locations

To establish the natural range of variability of metals concentrations in sediment, cores were retrieved from six floodplain lakes (Fig. 1). The lakes were chosen based on proximity to the Peace and Athabasca rivers to capture a natural archive of sediment supplied by river floodwaters. Lakes cored in the Peace sector include PAD 65, which is ~ 2 km from the Peace River, and PAD 67, which is north of the Peace sector and ~ 1 km from the Slave River. Because the Peace River contributes the majority of the water to the Slave River (English et al. 1997) and channels conveying outflow from the Athabasca sector experience flow reversals during ice-jams events that flood PAD 67 (e.g., Jasek 2019), the Peace River is likely the sole source of river water and suspended sediment entering PAD 67, despite its location downstream of the confluence with Lake Athabasca outflow. Sediment cores were collected from four flood-prone lakes in the Athabasca sector, including M7 (located ~ 1 km from the Athabasca River), PAD 32 (~ 6.5 km from the Embarras River, a distributary of the Athabasca River), and PAD 30 and PAD 31, which are *~* 0.07 km and ~ 0.2 km, respectively, from Mamawi Creek, a distributary that conveys Athabasca and Embarras river flow (Kay et al. 2019).

### Lake surface sediment sampling locations in 2017 and 2018

In September 2017, surface sediment samples were collected from 61 lakes that span the hydrological gradients of the Peace and Athabasca sectors of the PAD (Fig. 1). This dataset includes 38 lakes originally sampled by Wolfe et al. (2007) for hydrological and limnological characterization. Twenty-seven of the lakes are located in the Peace sector and 34 lakes are in the Athabasca sector. Lakes were categorized into sectors based on their geographic location. Lakes located north of PAD 37 (Jemis Lake - N58° 39′ 53.7″, W111° 27′ 12.4″; Fig. 1) are within the Peace sector and lakes to the south, including PAD 37, are within the Athabasca sector.

Ice-jam flooding occurred in the Peace and Athabasca sectors in late April and early May of 2018 (Jasek 2019), which provided excellent opportunity to acquire in July 2018 recently deposited river-supplied flood sediment from 20 flooded lakes (of the 61 lake set; Fig. 1) where it is known the flood layer of sediment was supplied from one of the Peace or Athabasca rivers. Mapping of floodwater extent in spring 2018 was determined from measurements of lake and river water isotope composition, specific conductivity, and observations of sampling personnel (Remmer et al. in press). Collection of surface sediment from the 20 flooded lakes (8 in the Peace sector, 12 in the Athabasca sector) carefully obtained only the clearly visible surficial flood layer of light gray inorganic river-supplied sediment at the top of the cores.

### Sediment core and surface sediment collection

Lake sediment cores analyzed in this study to develop the pre-1920 baselines were collected from an inflatable kayak or the floats of a helicopter using a Glew (1989) gravity corer (GC) from PAD 31 (GC-4, 38 cm long) in September 2010 and a hammer-driven gravity corer (HC) from PAD 32 (HC-3, 46 cm) in June 2015, PAD 65 (HC-1, 54 cm) and PAD 67 (HC-2, 56 cm) in June 2016, and PAD 30 (HC-1, 40 cm) and M2 (HC-1, 40 cm) in July 2016. Lake sediment cores were transported to a nearby field station, sectioned into 1-cm intervals using a vertical extruder (Glew 1988) and sealed in Whirl-Pak bags. Surface sediment samples were collected in September 2017 and July 2018 using a mini-Glew gravity corer (Glew 1991) deployed from a helicopter with floats. Surface sediments were typically collected from the center of each lake, as is the norm for paleolimnological studies, and consistent with locations of sediment cores used to develop the pre-1920 baselines. For each lake, surficial sediments were obtained from 3 to 4 deployments of the mini-Glew corer and combined into a Whirl-Pak bag to obtain a representative sample from the lake center location. In 2017, we collected the top ~ 1 cm of sediment. In 2018, we obtained only the uppermost flood layer of distinctive light gray inorganic-rich river-supplied sediment. All sediment samples were shipped on ice to the University of Waterloo and stored in a dark cold room (4 °C) until analysis.

### Radiometric dating of sediment cores

Radiometric dating of sediment cores was conducted at the University of Waterloo Environmental Research (WATER) Laboratory with the use of an Ortec co-axial HPGe Digital Gamma Ray Spectrometer (Ortec #GWL-120-15). Select 1-cm sediment intervals were freeze-dried and loaded into pre-weighed SARSTEDT polypropylene tubes to a height of 3.5 cm. The tubes were capped with a silicone disc, epoxy resin, and left for a minimum of 21 days, allowing ^222^Rn and its decay products to equilibrate with ^226^Ra prior to measuring activity of ^210^Pb, ^214^Bi, and ^214^Pb. Chronologies were developed using a Constant of Rate Supply (CRS; Appleby 2001) model, where the weighted mean of ^214^Pb and ^214^Bi activities were used to estimate supported ^210^Pb activities. Ages were calculated where unsupported ^210^Pb activity was present in the sample and a linear extrapolation using the calculated sedimentation rate from these measurements were applied to all depths below the presence of any unsupported ^210^Pb activity.

### Analysis of metals concentrations

Freeze-dried sediment from 1-cm intervals of the sediment cores and the lake surface sediment samples were disaggregated and homogenized using a mortar and pestle. Subsamples (~ 1 g) were submitted to ALS Canada Ltd. (Waterloo, Canada) for metals analysis following the method EPA 200.2/6020A, a partial digestion (using HNO_3_ and HCl) which liberates metals that may be environmentally available (US EPA 1998), as recommended by Birch (2017).

### RAMP/OSM river surficial sediment metals data

River-bottom sediment metals concentration data collected by RAMP and OSM from 2000 to 2015 were obtained from the RAMP (2018) database for seven sampling locations within the Athabasca sector of the PAD (ATR-ER, BPC-1, BPC-2, EMR-1, EMR-2, FLC-1, GIC-1; Fig. 1). River-bottom sediment was analyzed for metals concentrations following methods SW6010 (2000–2002), EPA 200.3/200.8-ICPMS (2003–2009), and EPA 200.2/6020A (2010–2015), as listed in RAMP and OSM annual reports (RAMP 2019). Metals concentrations in the river-bottom sediment samples obtained in 2010–2015 were analyzed using identical methods as the pre-1920 sediment core and lake surficial sediment samples.

### Numerical and statistical analyses

Pre-industrial baselines for sediment metals concentrations were established for the Peace River and the Athabasca River using the pre-1920 strata in the lake sediment cores as a framework to detect enrichment since onset of oil sands mining and production, following methods presented by Wiklund et al. (2012, 2014). Metals were normalized to account for mineralogical and granular variability in lake sediment. Metals preferentially adsorb to fine clay-sized grains (Loring 1991; Kersten and Smedes 2002; Birch 2017) and since many lakes in the PAD are subject to varying energy conditions, this can lead to variations in metals concentrations independent of any additional supply of metals due to anthropogenic activities. Therefore, positive linear correlations are expected between metals and normalizing agents, as metal concentrations should be proportionally linked to changes in grain size or mineralogy (Loring 1991). Using R (R Core Team 2018) and RStudio (RStudio Team 2016), Akaike’s Information Criterion with a correction for small sample size (AICc) was calculated to determine the best linear model for pre-1920 lake sediment metal concentrations and possible geochemical normalizers (Al, Li, Ti, Zr). Baselines were developed for all of the metals that possessed a significant (at alpha = 0.05) positive linear relation with Al concentration in the pre-1920 sediment samples (as a result of the AICc analysis—see below). Additionally, Al was utilized as the normalizing agent, rather than lithium (Li) as reported in Wiklund et al. (2014), to maximize the number of river sediment samples collected by RAMP and OSM that could be assessed on the pre-1920 baselines, as Li has only been reported by RAMP and OSM since 2010. Lakes used to form the baselines include PAD 65 and PAD 67 for the Peace sector and PAD 30, PAD 31, PAD 32, and M7 for the Athabasca sector. The range of natural variation for individual samples was established by constructing 95% Prediction Intervals (PI) around the pre-1920 baseline sediment metals concentrations relative to Al concentrations.

For each of the eight metals of interest, we ran a heterogeneity of slopes test to determine whether metal-Al linear relations differ for pre-1920 Peace versus Athabasca sector baselines. Heterogeneity of slopes tests were performed using the R “car” package (Fox and Weisberg 2019) in RStudio. For this, and all other subsequent statistical tests, alpha was set to 0.05. Lake surface sediment (2017, 2018) and RAMP/OSM river sediment (2000–2015) metals concentrations were plotted on sector-specific pre-1920 baselines, along with Canadian Council of Ministers of the Environment (CCME) Interim Sediment Quality Guidelines (ISQG) for the protection of aquatic life for Cd, Cr, Cu, Pb, and Zn (CCME 1999a), and Soil Quality Guidelines (SQG) for the protection of environmental and human health for Be, Ni and V (CCME) (1999b, 2015a, b), to evaluate the extent of anthropogenic enrichment. If metals concentrations plot within the bounds of the 95% PI, this was used to indicate a common source of metals between recently deposited sediments and sediments deposited prior to 1920. If > 2.5% of samples plot above the 95% PI, we considered this evidence of enrichment.

Enrichment factors (EF) were used to quantify the degree of enrichment of metals concentrations in surface sediment of lakes in the PAD collected in September 2017 and July 2018, relative to pre-1920 baseline metals concentrations. EFs are expressed as a ratio of the measured Al-normalized metal concentrations to the metal concentration that is expected based on its pre-1920 relationship with the normalizing agent (Al). Thus, an EF of 1.0 identifies no enrichment above baseline and EF of 2.0 identifies a doubling of metal concentration above baseline. Boxplots were used to present the distribution of EFs graphically.

A series of one-way analysis of variance (ANOVA) tests were conducted to determine if mean EF values differ significantly among the pre-1920 baseline samples, lake surface sediment samples collected in 2017 and lake surface sediment samples collected in 2018. For each metal, ANOVA tests were run separately for sites in the Peace sector and sites in the Athabasca sector. For tests which resulted in significant *p* values, post-hoc tests (Dunnett’s T3, which does not assume equal variances) were conducted to determine pairwise differences among the groups. To address the goals of this study, we focus on reporting results where there was significant enrichment above the pre-1920 baseline. The one-way ANOVA and post-hoc tests were performed using IBM-SPSS Statistics version 25.

To determine ability of the PAD lake surface sediment metals concentration data sets to detect a rise in metal EF values above the pre-1920 baseline, we ran a series of power analyses (one per metal for each of the four surface sediment datasets: Peace sector 2017 and 2018, and Athabasca sector 2017 and 2018) to (1) determine the minimum rise in EF (i.e., the effect size) that can be detected above an EF of 1.00 with 90% power and (2) calculate the power to detect a 10% rise in EF (i.e., rise in EF to 1.10; or, effect size of 0.10). The power analyses were run as one-sample tests at alpha = 0.05 and were one-tailed.

## Results

### Sediment core chronologies

**Fig. 2:**
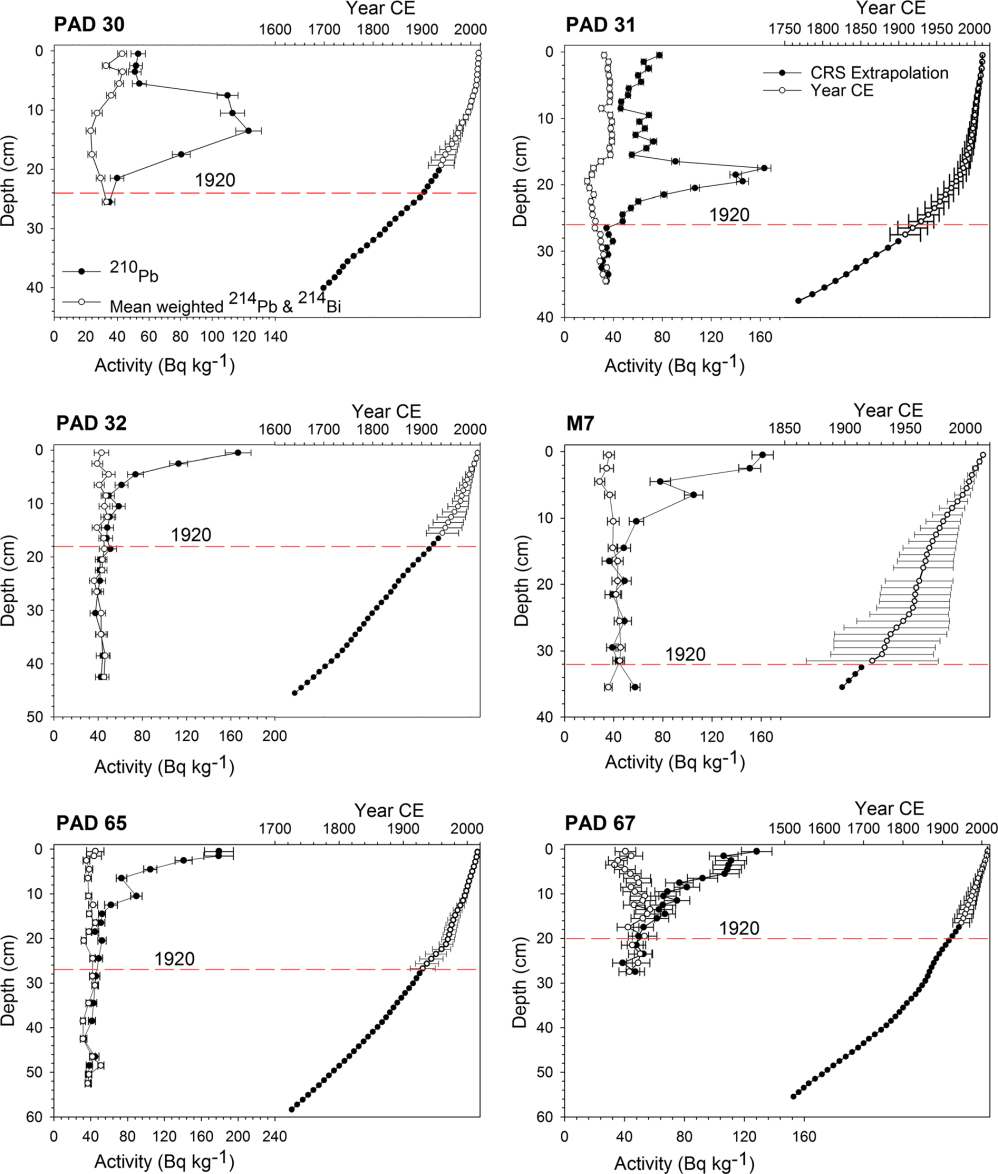
Activity profiles versus depth for ^210^Pb (closed circles) and mean weighted ^214^Pb and ^214^Bi (open circles) for sediment cores included in the pre-1920 baselines. Age-depth profiles for measured ^210^Pb (open circles) with error bars and extrapolated CRS model dates (closed circles)

Radiometric data were utilized to develop sediment core chronologies for the six lakes that defined pre-1920 metal concentrations (Fig. 2). ^210^Pb activity profiles in lake sediment cores from four lakes (PAD 32, M7, PAD 65, and PAD 67) show a relatively consistent decline of activity with depth. Background (i.e., supported) ^210^Pb activity for these four lakes was reached between 16 and 33 cm indicating variable sedimentation rates among lakes. For these lakes, pre-1920 sediments occur below 19, 32, 27, and 21 cm for PAD 32, M7, PAD 65, and PAD 67, respectively. Lakes PAD 30 and PAD 31 display different ^210^Pb activity profiles with low activity at the bottom of the core, a rise in activity at mid-depth, and a decline in the upper sections of the cores (Fig. 2). The decline in the top section of these cores correspond with increasing deposition of inorganic sediment related to the Embarras Breakthrough in 1982, an avulsion which conveyed Athabasca and Embarras river flows into Cree and Mamawi creeks and increased flooding at PAD 30 and PAD 31 (Kay et al. 2019). In the cores from PAD 30 and PAD 31, pre-1920 sediments occur below 24 and 26 cm, respectively.

### Developing pre-1920 baselines for sector-specific lakes

**Fig. 3:**
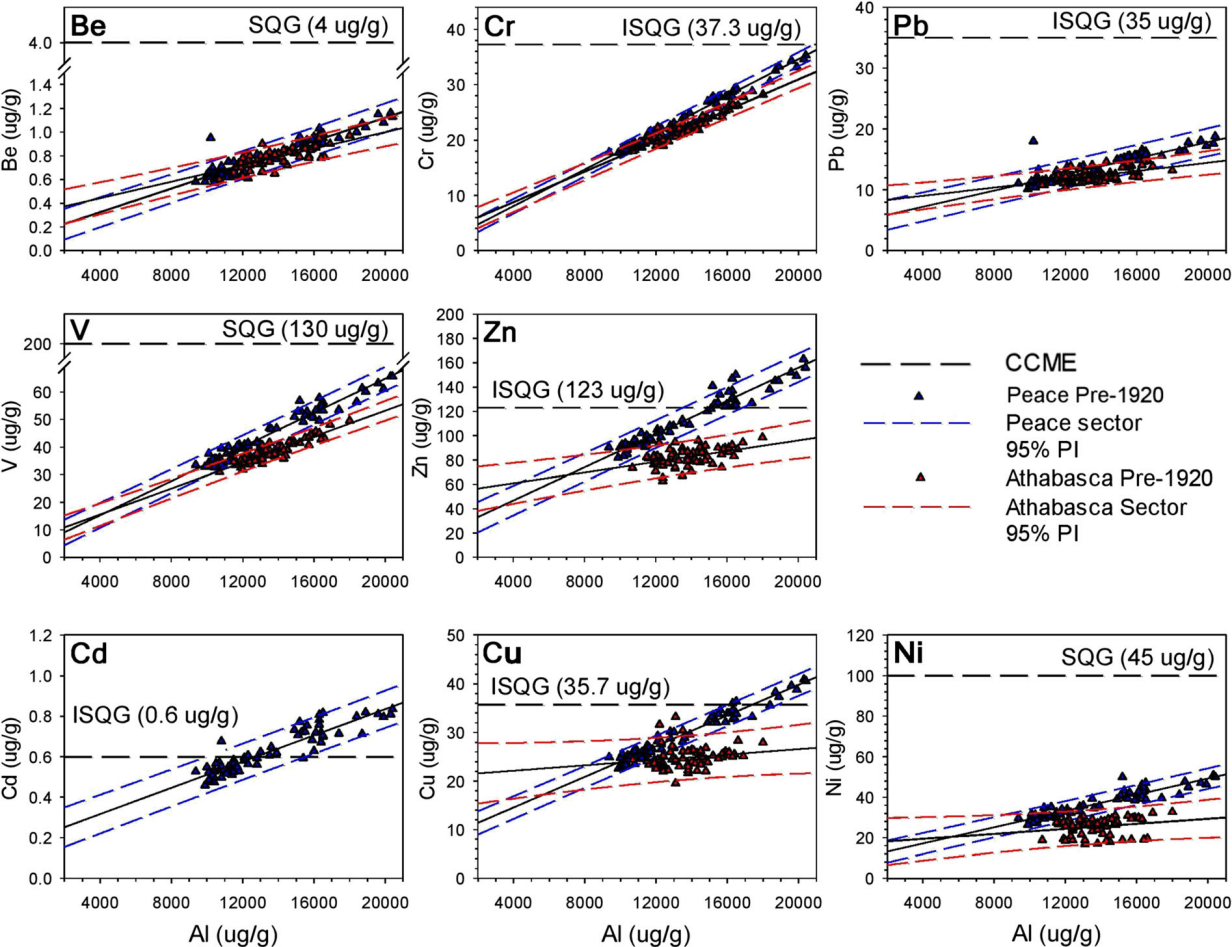
Cross-plots demonstrating the linear relations between pre-1920 metal concentrations and the normalizing agent (Al). The Peace River 95% PI (blue dashed lines) and regression lines are based on the pre-1920 measurements of metals from PAD 65 and PAD 67 (blue triangles). The Athabasca River 95% PI (red dashed lines) and regression lines are based on the pre-1920 measurements of metals concentrations from PAD 30, PAD 31, PAD 32, and M7 (red triangles). Canadian Council of Ministers of the Environment (CCME) (1999a, b, 2015a, b). Interim Sediment Quality Guidelines (ISQG) and Soil Quality Guidelines (SQG) plotted on *y*-axis denote the guideline concentrations for the metal of concern

Baseline linear relations, including 95% prediction intervals (PI), between the metals of interest (Be, Cd, Cr, Cu, Ni, Pb, V, Zn) and Al were developed, where possible, from measurements of the metals concentrations in pre-1920 sediment for the Peace and Athabasca sectors (Fig. 3). Analyses using AICc demonstrate that Al is the best normalizing agent for V in the Peace and Athabasca sectors (Table 1). Aluminum was also deemed the best normalizing agent for five of the other metals in both the Peace and Athabasca sectors (see Tables S1, S2). Statistically significant (at alpha = 0.05) positive linear relationships between metals (Peace sector: Be, Cd, Cr, Cu, Ni, Pb, V, and Zn; Athabasca sector: Be, Cr, Pb, V, and Zn) and Al indicate that these metals may be normalized using Al concentrations (Kersten and Smedes 2002; Table 2). Cadmium does not normalize in the Athabasca sector due to a negative slope with Al, which does not fit the criteria for normalization. Thus, the pre-1920 baseline for Cd is not shown in Fig. 3, or considered further. Relations between concentrations of Cu and Ni with Al are not significant in pre-1920 sediment core samples from the Athabasca sector. We consider these data in the heterogeneity of slopes tests, but are not used further to assess enrichment in lake and river surface sediment samples.

**Table 1:** Results of analysis using the Akaike information criterion with correction for small sample size (AICc) to determine the best pre-1920 normalizer model for vanadium in the Peace and Athabasca sectors. Results of AICc analysis for the other metals of interest are presented in Tables S1 and S2

Sector	Normalizer	AICc coefficient	Delta AICc	AICc weight
Peace	Al	280.62	0	1
	Li	344.86	64.24	5.20 × 10^–26^
	Zr	436.79	156.17	4.90 × 10^–28^
	Ti	455.12	174.5	2.59 × 10^–44^
Athabasca	Al	346.32	0	1
	Li	462.76	116.44	1.12 × 10^−14^
	Ti	472.09	125.77	1.22 × 10^−34^
	Zr	547.05	200.73	1.28 × 10^−38^

**Table 2:** Regression equations and *R*^2^ and *P* values for pre-1920 baselines metal-Al linear regressions for the Peace and Athabasca sectors. Statistically significant *P* values (at alpha = 0.05) are identified in italics

Sector	Metal	Regression equation	*R*^2^	*P* value
Peace	Be	*y* = 4.985 × 10^−5^*x* + 0.1244	0.88	*< 2.2 × 10*^*−16*^
	Cd	*y* = 3.242 × 10^−5^*x* + 0.1870	0.84	*< 2.2 × 10*^*−16*^
	Cr	*y* = 1.661 × 10^−3^*x* + 1.4117	0.99	*< 2.2 × 10*^*−16*^
	Cu	*y* = 1.579 × 10^−3^*x* + 8.2416	0.95	*< 2.2 × 10*^*−16*^
	Ni	*y* = 2.007 × 10^−3^*x* + 9.1078	0.87	*< 2.2 × 10*^*−16*^
	Pb	*y* = 6.660 × 10^−4^*x* + 4.5405	0.78	*< 2.2 × 10*^*−16*^
	V	*y* = 3.095 × 10^−3^*x* + 2.9212	0.95	*< 2.2 × 10*^*−16*^
	Zn	*y* = 6.823 × 10^−3^*x* + 19.3720	0.93	*< 2.2 × 10*^*−16*^
Athabasca	Be	*y* = 3.485 × 10^−5^*x* + 0.3024	0.52	*< 1.8 × 10*^*−11*^
	Cd	*y* = − 1.000 × 10^−5^*x* + 0.5025	0.04	0.129
	Cr	*y* = 1.387 × 10^−3^*x* + 3.2223	0.91	*< 2.2 × 10*^*−16*^
	Cu	*y* = 2.776 × 10^−4^*x* + 21.0130	0.04	0.129
	Ni	*y* = 6.217 × 10^−4^*x* + 16.9030	0.05	0.071
	Pb	*y* = 3.412 × 10^−4^*x* + 7.6384	0.28	*7.22 × 10*^*−06*^
	V	*y* = 2.352 × 10^−3^*x* + 6.1670	0.84	*< 2.2 × 10*^*−16*^
	Zn	*y* = 2.194 × 10^−3^*x* + 52.1310	0.22	*1.06 × 10*^*−4*^

Based on heterogeneity of slopes tests, slopes of all metal-Al relations are significantly steeper for the pre-1920 Peace sector regressions than those for the Athabasca sector (Tables 2 and 3; Fig. 3). Consequently, at higher Al concentrations, metals concentrations are elevated in the pre-1920 Peace sector baselines relative to those in the Athabasca sector baselines (Fig. 3). For the pre-1920 data from lakes in the Peace sector, several measured concentrations of Cd (48% of samples), Cu (13%), and Zn (38%) exceed the Canadian Council of Ministers of the Environment (CCME) Interim Sediment Quality Guidelines (ISQG) and concentrations for some samples of Ni (12.5% of samples) exceed Soil Quality Guidelines (SQG) (Fig. 3). No measured metals concentrations in pre-1920 sediment samples from lakes of the Athabasca sector exceed CCME guidelines.

**Table 3:** Results of a series of heterogeneity of slopes tests used to determine if the regression slopes differ between the Peace versus Athabasca sectors of the delta for the pre-1920 baseline metal-Al relations. The table presents type III sum of squares for the interaction term, degrees of freedom for sectors (Peace vs. Athabasca) and residuals (respectively), *F* test statistic, and *P* value for each metal investigated. Statistically significant *P* values (at alpha = 0.05) are identified in italics. Data for cadmium concentration were not analyzed because the slope of the relation with aluminum concentration was negative for the Athabasca sector (see text for further details)

Metal	Sum of squares	Degrees of freedom	*F* value	*P* value
Be	0.02767, 0.3855	1, 124	8.9	*0.003*
Cr	9.237, 52. 79	1, 124	21.7	*8.1 × 10*^*−6*^
Cu	208.08, 383.55	1, 124	67.27	*< 2.54 × 10*^*−13*^
Ni	236.1, 1461	1, 124	19.98	*1.74 × 10*^*−5*^
Pb	12.97, 121.8	1, 124	13.21	*4.07 × 10*^*−4*^
V	67.87, 429.2	1, 124	19.61	*2.06 × 10*^*−5*^
Zn	2635, 4653	1, 124	70.21	*< 9.73 × 10*^*−14*^

### Assessment of surface sediment metals concentrations from 2017 and 2018 relative to pre-1920 baselines

**Fig. 4:**
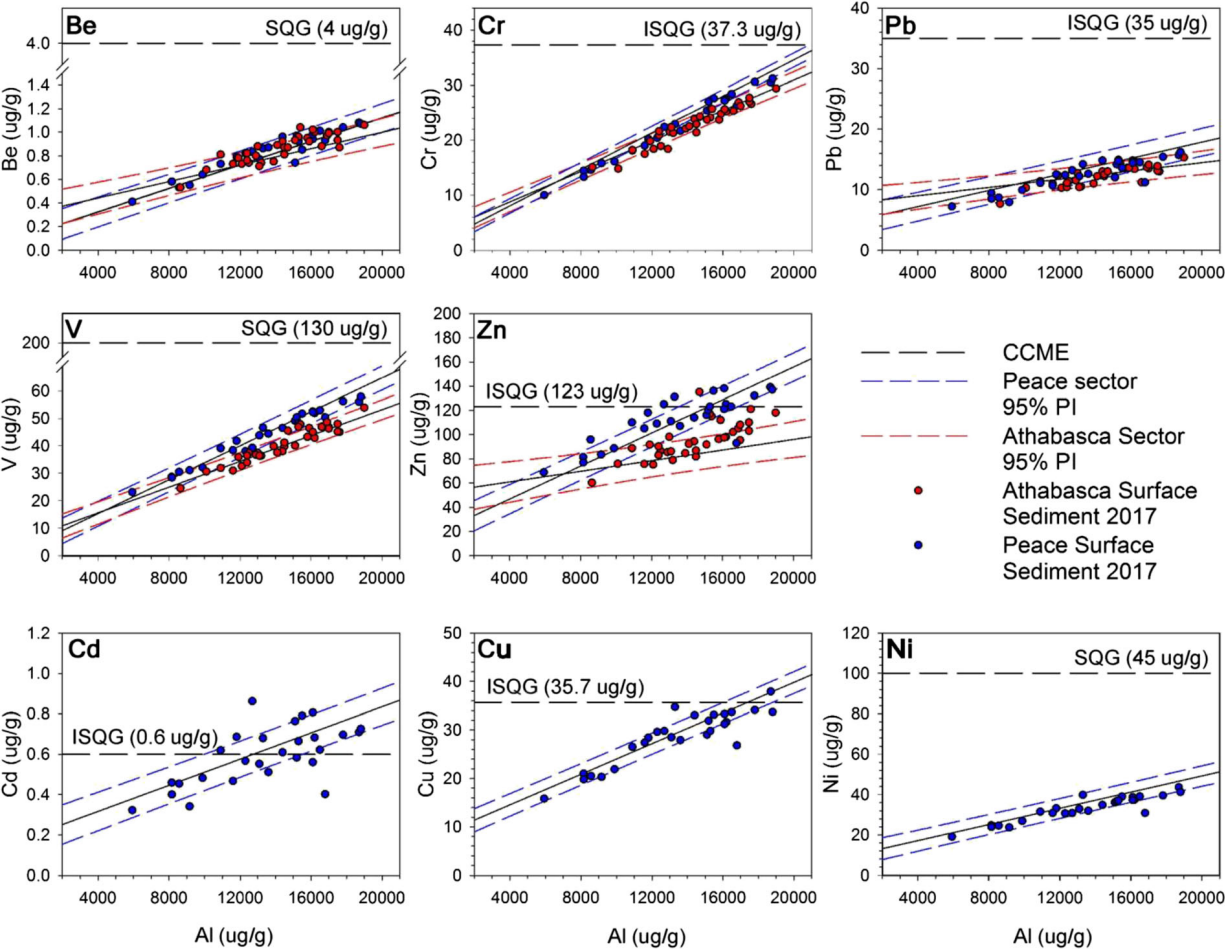
Metals concentrations from surface sediments (upper ~1 cm) collected in September 2017 from Peace sector lakes (blue circles) and Athabasca sector lakes (red circles) plotted on the pre-1920 linear regressions and 95% PIs. Canadian Council of Ministers of the Environment (CCME) (1999a, b, 2015a, b) Interim Sediment Quality Guidelines (ISQG) and Soil Quality Guidelines (SQG) plotted on *y*-axis denote the guideline concentrations for the metal of concern

**Fig. 5:**
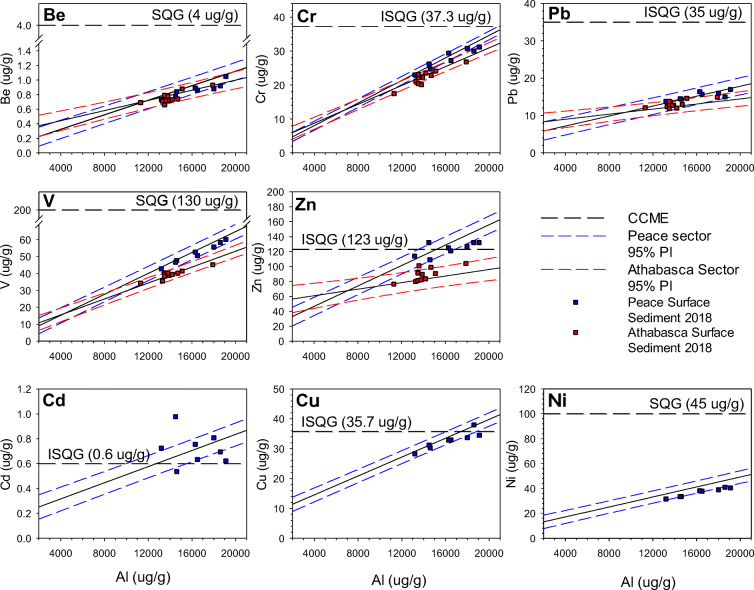
Metals concentrations from surficial flood-layer sediments collected in July 2018 after spring flooding from Peace sector lakes (blue squares) and Athabasca sector lakes (red squares) plotted on the pre-1920 linear regressions and 95% PIs. Canadian Council of Ministers of the Environment (CCME) (1999a, b, 2015a, b) Interim Sediment Quality Guidelines (ISQG) and Soil Quality Guidelines (SQG) plotted on *y*-axis denote the guideline concentrations for the metal of concern

Surficial lake sediment metals concentrations from 2017 (Fig. 4) and 2018 (Fig. 5) were assessed for evidence of enrichment relative to the pre-1920 baselines for the Peace and Athabasca sectors. Most metals concentrations from the 2017 dataset plot below the upper 95% PI (Fig. 4). This is entirely the case for Be, Cr, Ni, Pb, and V. For other metals, concentrations for some samples plot above the upper 95% PI. For the lakes in the Peace sector, this includes Cd (4 of 27 samples; 14.8%), Cu (1 of 27 samples; 3.7%), and Zn (5 of 27 samples; 18.5%). In the Athabasca sector, this includes Be (6 of 34 samples; 17.6%), Cr (1 of 34; 2.9%), Pb (1 of 34 samples; 2.9%), V (3 of 34 samples; 8.8%), and Zn (11 of 34 samples; 32.4%). Some measurements of Cd (14 of 27 samples; 51.9%), Cu (1 of 27 samples; 3.7%), and Zn (8 of 27 samples; 29.6%) exceed the CCME ISQG in the Peace sector, whereas one measurement of Zn (1 of 34 samples; 2.9%) exceeds the CCME ISQG in the Athabasca sector.

Similar patterns are evident for the metals concentrations in the 2018 flood-supplied surface sediment samples (Fig. 5). All measurements of Be, Cr, Ni, Pb, and V plot within the 95% PIs of the pre-1920 baselines, whereas concentrations of Cd and Zn exceed the 95% PIs in a few samples. In the Peace sector, these include 2 of 8 samples for Cd (25%) and 1 of 8 samples for Zn (12.5%). In the Athabasca sector, 2 of 12 samples (6.7%) plot above the upper 95% PI for Zn. Some metals concentrations in the 2018 surface sediment in the Peace sector exceed the CCME ISQG, including Cd (7 of 8 samples; 87.5%), Cu (1 of 8 samples; 12.5%), and Zn (5 of 8 samples; 62.5%).

### Assessment of RAMP/OSM river-bottom sediment metals concentrations on pre-1920 baselines

**Fig. 6:**
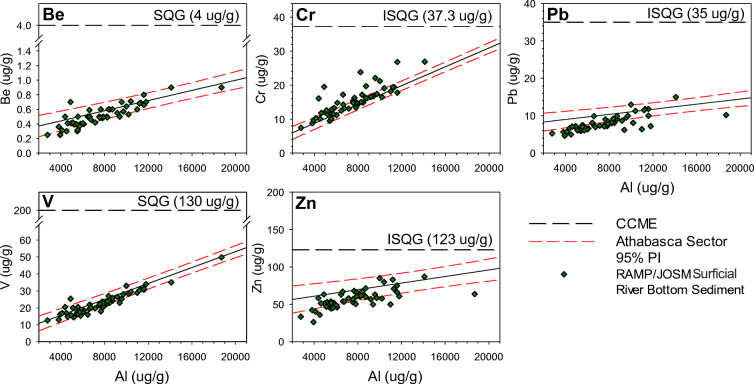
River-bottom sediment metals concentrations for samples collected by RAMP and OSM during 2000–2015 (RAMP 2019). Athabasca sector locations (ATR-ER, BPC-1, BPC-2, EMR-1, EMR-2, FLC-1, GIC-1) denoted by green diamonds plotted on the pre-1920 linear regressions and 95% PIs. Canadian Council of Ministers of the Environment (CCME) (1999a, b, 2015a, b) Interim Sediment Quality Guidelines (ISQG) and Soil Quality Guidelines (SQG) plotted on y-axis denoting the guideline concentrations for the metal of concern. Data are not shown for Cd, Cu, or Ni because pre-1920 relations with Al concentration are not statistically significant

River-bottom sediment metals concentrations measured in the Athabasca Delta by RAMP and OSM were assessed on pre-1920 baselines defined from Athabasca sector lake sediment cores, which include Be, Cr, Pb, V, and Zn (Fig. 6). The majority of RAMP/OSM river-bottom sediment samples plot closely along the pre-1920 baselines for Be, Pb, V, and Zn and within their 95% PIs. Some of the RAMP/OSM surficial river-bottom sediment metals concentrations are enriched relative to the upper limit of the pre-1920 lake-derived 95% PI for Be (1 of 51 samples; 2%), Cr (19 of 51 samples; 37.3%), Pb (2 of 51 samples; 3.9%), and V (1 of 51 samples; 2%). At low Al values, concentrations of Pb and Zn plot below pre-1920 baselines. No metal concentrations in the RAMP/OSM samples exceed CCME guidelines.

### Enrichment factor analysis

**Fig. 7:**
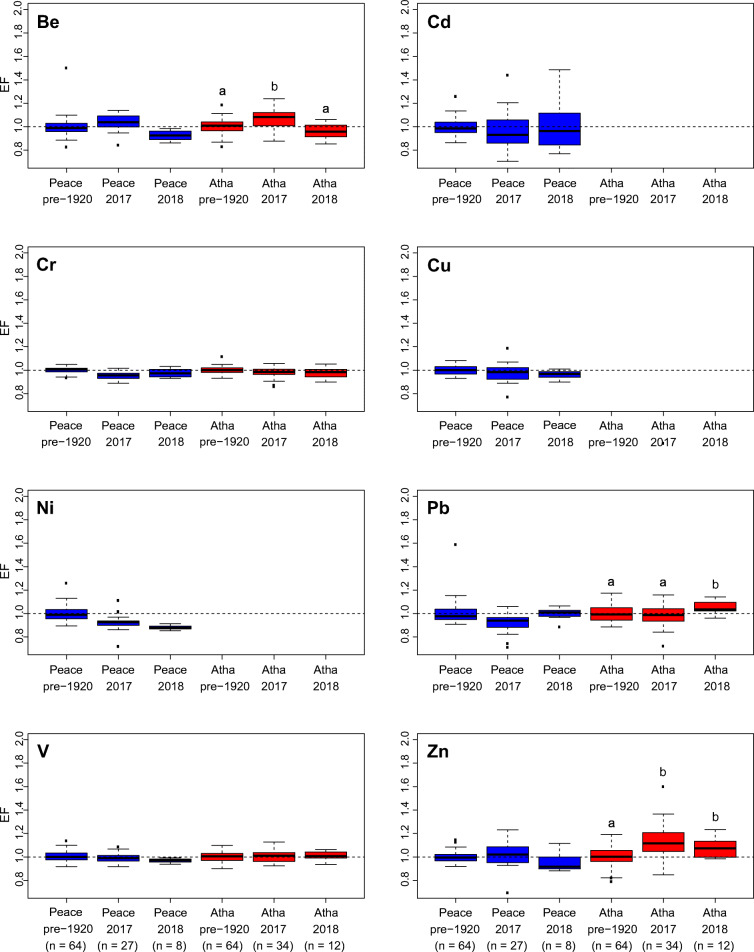
Boxplots showing the distribution of enrichment factors (EF) for metals (Be, Cd, Cr, Cu, Ni, Pb, V, and Zn) relative to sector-specific pre-1920 baseline concentrations. Enrichment factors were determined for Peace sector (PS; blue) surface sediment and Athabasca sector (AS; red) surface sediment from 2017 and 2018 sampling campaigns. Black dashed line at an EF value of 1.0 indicates no enrichment. Letters denote instances of significant (alpha = 0.05) increase in mean EF relative to pre-1920 baseline, as determined from one-way ANOVA and post-hoc tests

Enrichment factors (EF) were calculated as a means of quantifying and summarizing metals concentrations in recently deposited lake sediments relative to the natural range of variability captured by the pre-1920 baseline samples (Fig. 7). For most metals in surficial lake sediment, median EF values are close to 1.0, and interquartile (25–75th percentile) ranges are typically narrow and center near 1.0. However, there are a few exceptions. Interquartile ranges are above an EF of 1.0 for Be in the Peace and Athabasca sector 2017 samples, Pb in the Athabasca sector 2018 samples, and Zn in the Athabasca sector 2017 and 2018 samples. Overall, this analysis demonstrates that no metals exceed an EF of 1.6 in an individual sample and most EF values are ≤ 1.3. For individual samples, the highest EFs are for Cd (1.5) in 2018 within the Peace sector and Zn (1.6) in the Athabasca sector.

At the Athabasca sector, there are a few instances of significantly higher mean metals EFs in 2017 and/or 2018 surficial lake sediments relative to the pre-1920 baseline values, but the mean differences are < 0.15 (i.e., < 15% enrichment above baseline; Tables 4 and 5; Fig. 7). The ability to detect such small differences to be significant is due to low sample variance and, consequently, high statistical power (for most metals, > 99.9% power to detect a 10% enrichment (min. power = 74.66%; max. = > 99.99%); Table 6). Mean EF for Be is significantly higher by 0.07 in the 2017 samples compared to the pre-1920 baseline. For Pb, mean EF is significantly higher by 0.05 in the 2018 samples compared to the pre-1920 baseline. Mean EF for Zn is significantly higher in both the 2017 (0.13) and 2018 (0.08) samples compared to the pre-1920 baseline. In all other instances, there is no significant enrichment of metals concentrations above pre-1920 baselines.

**Table 4:** Results for a series of one-way ANOVA tests to determine if enrichment factors differ between sector-specific pre-1920 baselines and 2017 and 2018 surface sediments. Table presents the sum of squares and degrees of freedom (d.f.) for the treatment (pre-1920 baseline, 2017 surface sediment, 2018 surface sediment) and error (within-groups) components (respectively), and *P* values. Statistically significant *P* values (at alpha = 0.05) are identified in italics

Sector	Metal	Sum of squares	d.f. (treatment, error)	*F* value	*P* value
Peace	Be	0.081, 0.582	2, 96	6.7	*1.89 × 10*^*−3*^
	Cd	0.040, 1.494	2, 96	1.29	0.28
	Cr	0.048, 0.085	2, 96	27.0	*4.87 × 10*^*−10*^
	Cu	0.020, 0.264	2, 96	3.64	0.03
	Ni	0.176, 0.375	2, 96	22.6	*9.30 × 10*^*−9*^
	Pb	0.116, 0.721	2, 96	7.76	*7.55 × 10*^*−4*^
	V	0.007, 0.190	2, 96	1.78	0.178
	Zn	0.036, 0.528	2, 96	3.25	*0.04*
Athabasca	Be	0.152, 0.546	2, 107	14.9	*1.96 × 10*^*−6*^
	Cr	0.012, 0.156	2, 107	4.1	*0.02*
	Pb	0.043, 0.606	2, 107	3.81	*0.02*
	V	0.002, 0.215	2, 107	0.57	0.57
	Zn	0.406, 1.148	2, 107	18.9	*9.26 × 10*^*−8*^

**Table 5:** Results of post-hoc Dunnett T3 tests used to determine differences in sector-specific mean enrichment (mean EFs) among 2017 surface sediment (Peace sector d.f. = 26; Athabasca sector d.f. = 33), 2018 surface sediment (Peace sector d.f. = 7; Athabasca sector d.f. = 11) and pre-1920 baseline sediment samples (Peace sector d.f. = 63; Athabasca sector d.f. = 63). Table presents the sample means and *P* values. Statistically significant *P* values (at alpha = 0.05) are identified in italics

Sector	Metal	Comparison		Mean EF		*P* value
		Sample 1	Sample 2	Sample 1	Sample 2	
Peace sector	Be	Baseline	2017	1.00	1.04	0.076
		Baseline	2018	1.00	0.93	*0.004*
		2017	2018	1.04	0.93	*9.94 × 10*^*−5*^
	Cd	Baseline	2017	1.00	0.96	0.526
		Baseline	2018	1.00	1.01	0.998
		2017	2018	0.96	1.01	0.895
	Cr	Baseline	2017	1.00	0.95	*2.53 × 10*^*−7*^
		Baseline	2018	1.00	0.98	0.311
		2017	2018	0.95	0.98	0.306
	Cu	Baseline	2017	1.00	0.97	0.282
		Baseline	2018	1.00	0.96	0.075
		2017	2018	0.97	0.96	0.936
	Ni	Baseline	2017	1.00	0.92	*2.28 × 10*^*−5*^
		Baseline	2018	1.00	0.88	*1.65 × 10*^*−12*^
		2017	2018	0.92	0.88	*0.013*
	Pb	Baseline	2017	1.00	0.92	*0.001*
		Baseline	2018	1.00	1.00	0.999
		2017	2018	0.92	1.00	*0.021*
	V	Baseline	2017	1.00	0.99	0.841
		Baseline	2018	1.00	0.97	*0.006*
		2017	2018	0.99	0.97	0.114
	Zn	Baseline	2017	1.00	1.03	0.564
		Baseline	2018	1.00	0.95	0.377
		2017	2018	1.03	0.95	0.168
Athabasca sector	Be	Baseline	2017	1.00	1.07	*1.663 × 10*^*−4*^
		Baseline	2018	1.00	0.96	0.198
		2017	2018	1.07	0.96	*1.893 × 10*^*−4*^
	Cr	Baseline	2017	1.00	0.98	0.072
		Baseline	2018	1.00	0.98	0.294
		2017	2018	0.98	0.98	0.998
	Pb	Baseline	2017	1.00	0.98	0.579
		Baseline	2018	1.00	1.05	*0.046*
		2017	2018	0.98	1.05	*0.012*
	V	Baseline	2017	1.00	1.01	0.754
		Baseline	2018	1.00	1.01	0.840
		2017	2018	1.01	1.01	1.00
	Zn	Baseline	2017	1.00	1.13	*2.329 × 10*^*−5*^
		Baseline	2018	1.00	1.08	*0.029*
		2017	2018	1.13	1.08	0.272

**Table 6:** Results of power analysis for 2017 and 2018 lake surface sediment metals enrichment factor (EF) datasets from the Peace and Athabasca sectors to determine the statistical power to detect an enrichment of 10% (i.e., mean EF = 1.10) at alpha = 0.05, and to determine the sensitivity of the methods (i.e., effect size) to detect a 10% enrichment (i.e., mean EF = 1.10) above pre-1920 baseline with 90% power (i.e., 90% certainty of avoiding a type 2 error) and at alpha = 0.05 (i.e., 5% chance of a type 1 error). Power analyses were performed for each of the metals with significant positive linear relations with aluminum concentration in pre-1920 lake sediment core samples. Power was assessed for 2017 (Peace sector d.f. = 26; Athabasca sector d.f. = 33) and 2018 sediment samples (Peace sector d.f. = 7; Athabasca sector d.f. = 11)

Sector	Metal	Year	Variance (EF units)^2^	Power (%)	Effect size (EF %)
Peace sector	Be	2017	0.005	> 99.99	4
		2018	0.002	99.85	5
	Cd	2017	0.031	88.79	10
		2018	0.056	74.66	28
	Cr	2017	0.001	> 99.99	2
		2018	0.002	99.85	5
	Cu	2017	0.006	99.98	5
		2018	0.001	99.99	4
	Ni	2017	0.004	> 99.99	4
		2018	0.000	> 99.99	2
	Pb	2017	0.007	99.94	5
		2018	0.003	99.32	6
	V	2017	0.002	> 99.99	3
		2018	0.000	> 99.99	2
	Zn	2017	0.013	99.58	7
		2018	0.007	90.95	10
Athabasca sector	Be	2017	0.007	> 99.99	4
		2018	0.004	99.82	6
	Cr	2017	0.002	> 99.99	2
		2018	0.002	> 99.99	4
	Pb	2017	0.008	> 99.99	5
		2018	0.003	99.96	5
	V	2017	0.003	> 99.99	3
		2018	0.002	> 99.99	4
	Zn	2017	0.020	98.97	7
		2018	0.007	98.06	8

Note that EFs are not shown for the RAMP/OSM data because baseline metals concentrations at lower Al values (~ 4000–10,000 μg/g) are not adequately characterized for river-bottom sediment. Since many RAMP/OSM metals concentrations plot below the pre-1920 regression lines, most of the EF interquartiles (25–75th percentile) are below 1.0, the implications of which are discussed further below.

## Discussion

Use of floodplain lake sediment cores to establish sector-specific pre-1920 baseline metals concentrations in the PAD

Lack of knowledge of the natural range of variation in sediment metals concentrations has long hampered ability to accurately assess for evidence of metals enrichment in the Athabasca River by oil sands operations (Schindler 2010). To address this, paleolimnological approaches were used in this study to establish pre-industrial (defined as pre-1920; see Wiklund et al. 2012, 2014) baseline sediment metals concentrations from flood-prone lakes in both the Peace and Athabasca sectors of the PAD at the terminus of the Athabasca River.

Comparison of baselines established from Al-normalized metals concentrations in sediments deposited before 1920 demonstrates that Peace River-sourced sediment possessed significantly steeper metal-normalizer relations, resulting in some elevated metals concentrations relative to the Athabasca sector, especially for Cu and Zn, but also V—a key metal of concern related to oil sands development (Tables 2 and 3; Fig. 3). This likely can be attributed to the geological differences of their respective drainage basins. For example, the Peace River flows over and alongside several fault lines near the town of Peace River, Alberta (Rukhlov 2011). Faults can act as pathways for upwelling of mineralized fluids and, as a result, several sedimentary exhalative deposits (rich in Cu, Pb, and Zn) and black shale polymetallic deposits (rich in Ni and Zn) have been reported (Rukhlov 2011). These deposits are likely eroded by the Peace River and incorporated into the natural river sediment load, increasing concentrations of Cu, Ni, Pb, and Zn relative to sediment conveyed by the Athabasca River. Additionally, the Peace River flows through ~ 10 km of Devonian carbonate outcrops, which are located along the riverbank between the upper and lower Vermilion Chutes. Alberta Geological Survey reports have identified that these carbonate outcrops contain relatively high concentrations of Zn (Rice 2003; Pana 2003). It has been reported that Cd is often an element or impurity associated with Zn ores (Schwartz 2000). Therefore, the Zn-rich Vermilion Chutes outcrop may explain why natural concentrations of Cd and Zn plot above CCME ISQG in lakes of the Peace sector (Fig. 3). Notably, concentrations of Ni and V are higher in the pre-1920 Peace sector baselines compared to the Athabasca sector baselines, despite the Athabasca River flowing through the bitumen-rich McMurray Formation, which contains relatively high concentrations of Ni and V (Speight 2005).

Failure to account for these distinct differences in sediment metals concentrations supplied via the Peace River versus the Athabasca River can lead to erroneous conclusions about enrichment of metals from oil sands development. For example, if sediment supplied by the Peace River to floodplain lakes in the PAD were evaluated on the pre-1920 baseline of the Athabasca sector lake sediment cores, many of the samples would be erroneously identified as being enriched. While some of these metals (Cd, Cu, and Zn) possess concentrations above CCME ISQG in both the 2017 and 2018 datasets for the Peace sector, metals concentrations indeed fall within the range of natural variation defined for the sediment conveyed by the Peace River. Geological deposits listed above provide just a few of the potential sources that may lead to differences in the observed metals concentrations in lake sediment supplied by the Peace and Athabasca rivers. Further analysis of source contribution of metals upstream is required to more fully explain the differing metal-Al relations observed in this study.

The six collective lake sediment cores used to establish pre-1920 baseline metals concentrations span a broad range (~ 10,000–18,000 μg/g) of Al concentrations used to characterize metal-Al relations in lake sediment (Fig. 3). This range is adequate to capture the Al concentrations sampled from surficial sediments collected in 2017 from lakes spanning the hydrological gradient in the PAD and the 2018 flood-derived deposits. For the Athabasca sector, however, metal-Al relations are weak for Cd, Cu, and Ni, which prevented assessment of enrichment of these metals in the surface sediments (Table 2). Analysis of additional lake sediment cores may improve ability to incorporate Cd, Cu, and Ni into future surface sediment assessments. Fortunately, relations are strong for V, a metal of concern with respect to oil sands development.

### Comparison of 2017 and 2018 surficial lake sediment datasets to pre-1920 baselines

Analysis of two surficial lake sediment datasets provided an effective approach to assess the degree of metals enrichment across the ~ 6000 km^2^ PAD. The 2017 dataset included ~ 1 cm thick surficial sediments obtained from 61 lakes that span the full range of hydrological conditions (Wolfe et al. 2007). In this dataset, only Be and Zn EFs in the Athabasca sector are significantly higher (by 7 and 13%, respectively; Table 5) than the pre-1920 baseline. Sedimentation rates differ substantially across this hydrological gradient; thus, the time intervals captured by those samples vary among lakes, which may include flood and non-flood conditions. In contrast, the 2018 dataset captures a flood event and represents a snapshot of metals concentrations in river sediment conveyed by spring floodwaters. As suggested by Kelly et al. (2010), metals accumulated over winter months in the snowpack in the oil sands region (Kirk et al. 2014) become mobilized during the spring freshet and may move from the landscape to the Athabasca River. Thus, the 2018 surficial spring-flood layers have stronger ability to detect metals enrichment by oil sands operations than the 2017 surficial lake sediment dataset. Despite this, one-way ANOVA tests demonstrated that EFs are not significantly elevated in 2018 flood-derived sediment compared to pre-1920 baseline, with the exceptions of very small enrichment of Pb (5%) and Zn (8%) (Table 5). Collectively, the spatially comprehensive 2017 dataset along with the opportunistic sampling of the 2018 spring-flood deposits provide strong evidence that oil sands development has not yet caused marked enrichment of metals in recently deposited lake sediment relative to the pre-1920 baselines. We note that other studies have utilized an EF threshold of 1.5 to distinguish pristine conditions from those affected by human activities (e.g., Birch 2017) and > 99% of all individual surface sediment samples in the Athabasca sector measured in our study possessed an EF < 1.5.

### Assessing vanadium concentrations in surficial lake sediments in the PAD

Vanadium was scrutinized because it has been identified as an oil sands indicator metal for contamination (Gosselin et al. 2010; Wiklund et al. 2014) and is elevated in aerial deposition surrounding mining and bitumen processing activities (Kirk et al. 2014; Cooke et al. 2017; Klemt 2018). Cooke et al. (2017) demonstrated from analyses of lake sediment cores that enrichment of V via aerial pathways remains clearly detectable at near- (~ 8x background) and mid-field (~ 3x background) upland lakes, relative to baseline concentrations. Atmospheric deposition of V (and other metals) may be a more important pathway to lakes within a 50-km radius of the oil sands development than transport by river floodwaters. Results reported by Klemt (2018) reinforced this notion, as analyses of sediment cores from floodplain lakes along the Athabasca River adjacent to oil sands mining and processing facilities demonstrated that enrichment of V was detected in weakly flood-influenced sediment indicative of aerial deposition, but was not enriched in river-supplied lake sediment. Similarly, river-supplied sediment to Athabasca sector lakes in 2018 demonstrated no significant enrichment (mean V EF of 1.00 ± 0.04, 1 SD; range 0.9–1.1) relative to pre-1920 concentrations (Table 5; Fig. 7) in spite of strong sensitivity revealed by power analyses. For example, the methods used can detect a rise in V EF as small as 10% with > 99% certainty for all lake surface sediment datasets. Thus, while near- to mid-field (0–50 km) atmospheric-sourced contamination is clearly detectable from oil sands operations (Kirk et al. 2014; Cooke et al. 2017; Klemt 2018), there is accumulating evidence of no far-field or downstream enrichment of V in sediment conveyed by the Athabasca River (Wiklund et al. 2014; Klemt 2018; Kay et al. in press; *this study*).

### Assessing for metals pollution in river-bottom sediments in the Athabasca sector of the PAD

The establishment of pre-1920 baseline metals concentrations constructed from lake sediment cores enabled cursory assessment of 15 years of RAMP and OSM river sediment samples in the Athabasca sector (Fig. 6). This included an additional 10 years that were not assessed by Wiklund et al. (2014) due to limitations of the selected normalizing agent (i.e., lithium). Analysis of these 15 years of data resulted in no significant elevated concentrations measured in most metals relative to pre-1920 concentrations (based on comparison with 95% PIs for metal-Al relations), consistent with the findings reported by Wiklund et al. (2014). However, a substantial number of samples (36 of 51; 70.6%) were enriched in Cr relative to pre-1920 concentrations (Fig. 6). The source of this enrichment in Cr is unknown.

Although our data indicate no enrichment of metals (except Cr) in the RAMP/OSM samples (2000–2015), there are challenges to interpreting these results. Our assessments of enrichment of the RAMP/OSM data are inhibited by what appears to be steeper metal-Al relations at the low Al concentrations typical of river-bottom sediment, which are outside the range of our lake-derived pre-1920 baseline concentrations. This would imply that our baselines for the lower Al concentrations may be over-estimating natural river-bottom metals concentrations (leading to apparent EF values below 1), which is evident for many of the metals. Clearly, these results demonstrate that baselines constructed from lake sediment in this study are best applied to lake sediment accumulated after industrial development, as opposed to the unknown time-frame represented by river-bottom sediment. Therefore, we advocate that sampling the fine-grained fraction of flood sediment deposited into floodplain lakes is a more sensitive, real-time approach for assessing metals concentrations of river-derived suspended sediment.

## Conclusions and recommendations

Scientific research is needed to inform environmental monitoring, and here, clearly defined research objectives have provided direction for future aquatic monitoring efforts using sediment from floodplain lakes in the PAD. In the absence of long-term monitoring data, the use of paleolimnological approaches has demonstrated to be an effective method in characterizing natural concentrations of metals in sediments from flood-prone lakes, which are required to assess contemporary metals concentrations (also see Birch 2017). Results demonstrate no substantial enrichment of metals concentrations derived from oil sands operations in recently deposited sediment in lakes of the PAD. This includes samples collected in 2018, exclusively conveyed by river floodwaters during the spring freshet. The lack of marked enrichment in these samples, including V (an oil sands indicator metal), provides strong evidence that substantial metals contamination by oil sands development is not detectable in sediments of flood-prone lakes in the PAD.

The foundation of a successful sediment quality monitoring program requires knowledge of pre-development baseline metals concentrations and natural variation to accurately detect environmental changes due to anthropogenic activities (Smol 1992; Lindenmayer and Likens 2009; Dowdeswell et al. 2010; Wrona and di Cenzo 2011). The development of this framework using floodplain lake sediment cores to construct pre-1920 metal-aluminum linear relations was intended to build the foundation for continued aquatic sediment monitoring in the PAD, and could serve as a contribution to implementing Wood Buffalo National Park’s Action Plan (WBNP 2019). An important discovery is the need for Peace and Athabasca sector-specific baselines due to different metal-aluminum linear relations for sediment supplied by the Peace versus Athabasca rivers. The full 61-lake dataset is spatially comprehensive, spans the range of hydrological conditions and might be considered to be re-sampled regularly (e.g., every 5 years) to track sediment metals concentrations, given that oil sands operations propose to expand closer to the PAD. Metals concentrations exceeded CCME guidelines only in the Peace sector lakes, including in sediments deposited before 1920, a finding that implies future potential contamination of anthropogenic sources could have greatest effects in those lakes. Because contamination from the oil sands via the Athabasca River is a major concern, it may be preferential to sample lakes in the Athabasca sector more frequently (e.g., every 2–3 years). If coupled with routine water isotope monitoring of hydrological conditions (see Remmer et al. in press), then opportunistic lake surface sediment sampling should be conducted soon after flood events to capture freshly deposited river-supplied sediment, as was done in July 2018, to provide a snapshot of river sediment metals concentrations at the time of the spring freshet. Sampling river-borne sediment for metals at this time is important because this is when contaminants accumulated in the winter snowpack are discharged to the rivers, when metals are likely most toxic due to decline of water hardness, and when concerns are high for many biota at vulnerable, early life stages (Kelly et al. 2009, 2010).

## Electronic supplementary material

ESM. 1 (DOCX 20 kb)
