# Supplementary material for: Use of pre-industrial baselines to monitor anthropogenic enrichment of metals concentrations in recently deposited sediment of floodplain lakes in the Peace-Athabasca Delta (Alberta, Canada)
Source: Environ Monit Assess. 2020 Jan 10;192(2):106. doi: 10.1007/s10661-020-8067-y (PMC6954137; doi:10.1007/s10661-020-8067-y)
Supplement: Supplementary file 1 — (DOCX 20 kb) [file 10661_2020_8067_MOESM1_ESM.docx]

**Supplementary Information**

**Table S1**. Results of analysis using the Akaike information criterion with correction for small sample size (AICc) to determine the best geochemical normalizer for pre-1920 sediment in the Peace sector of the Peace-Athabasca Delta for the metals of interest.

| Metal of Concern | Normalizer | AICc coefficient | Delta AICc | AICc weight |
| --- | --- | --- | --- | --- |
| Be | Li | -255.258 | 0 | 1 |
|  | Al | -177.328 | 77.9301 | 1.20x10^-17^ |
|  | Ti | -98.7436 | 156.5146 | 1.03x10^-34^ |
|  | Zr | -59.9762 | 195.2819 | 3.94x10^-43^ |
| Cd | Al | -213.0188 | 0 | 1 |
|  | Li | -195.7156 | 17.3033 | 17.4x10^-4^ |
|  | Zr | -120.5578 | 92.4611 | 8.36x10^-21^ |
|  | Ti | -109.1544 | 103.8645 | 2.79x10^-23^ |
| Cr | Al | 122.8169 | 0 | 1 |
|  | Li | 252.1387 | 129.3218 | 8.28x10^-29^ |
|  | Zr | 352.8972 | 230.0803 | 1.09x10^-50^ |
|  | Ti | 373.7033 | 250.8863 | 3.32x10^-55^ |
| Cu | Al | 195.6049 | 0 | 1 |
|  | Li | 263.6881 | 68.0833 | 1.64x10^-15^ |
|  | Zr | 350.6677 | 155.0628 | 2.13x10^-34^ |
|  | Ti | 378.5462 | 182.9413 | 1.88x10^-40^ |
| Ni | Al | 299.9726 | 0 | 1 |
|  | Li | 323.9603 | 23.9877 | 6.18x10^-6^ |
|  | Zr | 392.5817 | 92.6090 | 7.77x10^-21^ |
|  | Ti | 417.2645 | 117.2918 | 3.39x10^-26^ |
| Pb | Li | 170.8444 | 0 | 1 |
|  | Al | 198.4352 | 27.5908 | 1.02x10^-6^ |
|  | Zr | 232.8280 | 61.9836 | 3.47x10^-14^ |
|  | Ti | 278.7717 | 107.9273 | 3.66x10^-24^ |
| V | Al | 280.6179 | 0 | 1 |
|  | Li | 344.8625 | 64.2446 | 5.20x10^-26^ |
|  | Zr | 436.7883 | 156.1704 | 4.90x10^-28^ |
|  | Ti | 455.1171 | 174.4992 | 2.59x10^-44^ |
| Zn | Al | 407.2074 | 0 | 1 |
|  | Li | 450.7297 | 43.5222 | 3.54x10^-10^ |
|  | Zr | 547.0623 | 139.8548 | 4.27x10^-31^ |
|  | Ti | 570.0306 | 162.8231 | 4.40x10^-36^ |

**Table S2**. Results of analysis using the Akaike information criterion with correction for small sample size (AICc) to determine the best geochemical normalizer for pre-1920 sediment in the Athabasca sector of the Peace-Athabasca Delta for the metals of interest.

| Metal of Concern | Normalizer | AICc coefficient | Delta AICc | AICc Weight |
| --- | --- | --- | --- | --- |
| Be | Li | -255.258 | 0 | 0.9267 |
|  | Al | -177.328 | 77.9301 | 0.0732 |
|  | Ti | -98.7436 | 156.5146 | 3.48x10^-8^ |
|  | Zr | -59.9762 | 195.2819 | 1.97x10^-15^ |
| Cd | Al | -213.0188 | 0 | 0.5491 |
|  | Li | -195.7156 | 17.3033 | 0.2673 |
|  | Zr | -120.5578 | 92.4611 | 0.1562 |
|  | Ti | -109.1544 | 103.8645 | 0.0274 |
| Cr | Al | 122.8169 | 0 | 1 |
|  | Li | 252.1387 | 129.3218 | 5.55x10^-37^ |
|  | Zr | 352.8972 | 230.0803 | 9.65x10^-38^ |
|  | Ti | 373.7033 | 250.8863 | 6.10x10^-55^ |
| Cu | Al | 195.6049 | 0 | 0.9533 |
|  | Li | 263.6881 | 68.0833 | 0.0177 |
|  | Zr | 350.6677 | 155.0628 | 0.0145 |
|  | Ti | 378.5462 | 182.9413 | 0.0145 |
| Ni | Al | 299.9726 | 0 | 1 |
|  | Li | 323.9603 | 23.9877 | 2.31x10^-6^ |
|  | Zr | 392.5817 | 92.6090 | 7.33x10^-7^ |
|  | Ti | 417.2645 | 117.2918 | 1.04x10^-8^ |
| Pb | Li | 170.8444 | 0 | 1 |
|  | Al | 198.4352 | 27.5908 | 2.75x10^-6^ |
|  | Zr | 232.8280 | 61.9836 | 4.54x10^-7^ |
|  | Ti | 278.7717 | 107.9273 | 6.03x10^-11^ |
| V | Al | 280.6179 | 0 | 1 |
|  | Li | 344.8625 | 64.2446 | 1.12x10^-14^ |
|  | Zr | 436.7883 | 156.1704 | 1.22 x10^-34^ |
|  | Ti | 455.1171 | 174.4992 | 1.28 x10^-38^ |
| Zn | Al | 407.2074 | 0 | 1 |
|  | Li | 450.7297 | 43.5222 | 4.21x10^-5^ |
|  | Zr | 547.0623 | 139.8548 | 1.14x10^-7^ |
|  | Ti | 570.0306 | 162.8231 | 2.25x10^-8^ |
